# Opportunities and Challenges for Health-Promoting Interventions for Older Adults: A Focus Group Study with Professionals in Municipal Health and Social Care

**DOI:** 10.1007/s44474-026-00008-0

**Published:** 2026-09-15

**Authors:** Linnea Körlof, Anneli Nyman, Ellinor Larsson, Gunilla Isaksson

**Affiliations:** Department of Health, Education and Technology, Luleå tekniska universitet, Luleå, Sweden

**Keywords:** Health promotion, Older adults, Occupational therapy, Organizational readiness, Municipal health, Social care

## Abstract

**Background:**

An aging population and an ongoing transition toward person-centered and integrated care highlight the need for health-promoting interventions for older adults, requiring new approaches within municipal health and social care.

**Aim:**

The aim of this study was to explore and describe how professionals in municipal health- and social care discuss opportunities and challenges within their organization related to health-promoting interventions directed at older adults.

**Material and Methods:**

A qualitative focus group design was used. Twenty-two participants, including occupational therapists, development managers, area managers, operations managers, and case managers from seven municipalities, participated in five focus group discussions. Data were analyzed inductively using a focus group analysis approach.

**Results:**

One overarching theme was identified: *Creating organizational readiness for health-promoting interventions*, comprising three subthemes: *Supportive structures for health-promoting work within the organization are needed*, *A shared health-promoting focus and new collaborations are required*, and *New working methods are necessary to facilitate health promotion.* These subthemes are interrelated together dynamically shape organizational readiness for health-promoting interventions.

**Conclusions and Significance:**

Taken together, The results indicate that organizational readiness within municipal health and social care is currently insufficient for health-promoting interventions to permeate practice yet has inherent potential to be actively created. Creating such readiness therefore requires coordinated, long-term efforts across the organization. For occupational therapy, these results underscore the importance of engaging in ongoing dialogue, both within the profession and across organizational levels, on how their competencies can be utilized and translated from reactive into more proactive and health-promoting approaches into everyday practices.

## Introduction

To manage future healthcare demands, society and healthcare systems worldwide need to address the challenges associated with the demographic trends of an aging population [1]. In response to these challenges, the European Commission [2] emphasizes collaboration in the development of sustainable health-promoting solutions that support older adults’ active and healthy ageing. Consequently, there is an ongoing transition in Swedish healthcare towards person-centered and integrated care, which emphasizes proactive and health-promoting approaches, as well as the utilization of digital solutions to provide more equal, efficient, and accessible healthcare services [3]. Swedish primary care constitutes the hub of this transition [3] and thus plays a vital role in driving these health-promoting approaches. Responsibility for primary healthcare in Sweden is shared between regions and municipalities and includes nonacute care that does not require hospital admission [4]. Municipal healthcare is provided in individuals’ own homes or in specialized housing and includes services ranging from simple one-time interventions to more extensive daily care and rehabilitative services [5]. The largest group (up to 83% in 2023) receiving municipal healthcare consists of individuals aged 65 or older [6]. Older adults in need of municipal healthcare often also receive municipal social services, such as home care. Staff in these services frequently perform delegated healthcare tasks, which can result in blurred boundaries between sectors [5]. Shared perspectives and collaborative efforts between these sectors are thus important in the transition towards more preventive and health-promoting approaches for older adults.

Research suggests that older adults generally have higher levels of physical and mental functioning than previous generations [7–9]. In Sweden, improvements in health, longevity, and the ability to perform activities among older adults up to 80 years of age have also been reported [10]. The process of enabling people to increase control over the determinants of health, and thereby improve their health, is defined as health promotion [11]. This indicates that health-promoting and preventive interventions targeting key health determinants among community-dwelling older adults, before they enter municipal health and social care, may help maintain health and delay the onset of care needs [12]. Such a shift in practice could reduce future demand on health and social care systems.

In line with the ongoing transition to person-centered and integrated care [3], there is a need to expand occupational therapy practices towards preventive and population-based approaches.

[8]. Within occupational therapy, engagement in meaningful activities of everyday life is considered a key determinant of health [14], highlighting its potential contribution to health-promoting interventions. Moreover, demographic changes and increasing demands on municipal health and social care require the development of new and more efficient ways of delivering such interventions. The growing digitalization of society [15] and evidence supporting group-based approaches [16, 17] suggest that combining digital delivery and group-based formats may offer promising ways to support health promotion among older adults. These developments underscore the need to explore how such interventions can be introduced and taken into practice within existing municipal health and social care systems. In response to this need, we saw the value of developing a digital group-based occupational therapy intervention to support healthy ageing [18], which was subsequently piloted on a small scale in a municipality healthcare context [19].

Occupational therapists are, however, part of a complex system involving diverse health and social care professionals across multiple organizational levels. Moreover, the transition towards person-centered and integrated care affects the entire municipal organization. Consequently, municipal area and development managers responsible for systematic quality improvements, leaders at multiple organizational levels within municipal healthcare and professionals within social care, collaborate with each other and with occupational therapists, thereby shaping their working conditions [4, 20]. Introducing new health-promoting occupational therapy interventions is thus a collateral concern within municipality health and social care. Previous findings, which do not include the northernmost county of Sweden, indicate that municipal health and social care in Sweden vary in terms of their organizational conditions and readiness to implement preventive and health-promoting approaches [21]. Despite the increasing emphasis on such approaches, limited attention has been given to how professionals across municipal health and social care understand and navigate the organizational conditions that shape their integration into practice. Understanding these conditions prior to implementation is emphasized in frameworks for process evaluation of complex interventions, where context is considered central [22], as well as in guidance on early-phase research highlighting the importance of exploring contextual conditions and stakeholder perspectives before more structured implementation frameworks are applied [23]. Thus, rather than focusing on intervention implementation per se, there is a need to better understand professionals’ perspectives on the organizational conditions that constitute the prerequisites for such integration into practice.

## Aim

The aim of this study was to explore and describe how professionals in municipal health and social care discuss opportunities and challenges within their organization related to health-promoting interventions directed at older adults.

## Materials and Methods

### Design

This study adopted a qualitative design with a focus group methodology, in which the generation of data enables the stimulation of dialog and interaction between participants, allowing them to draw on and develop each other’s perspectives [24]. This can provide a collective understanding of the opportunities and challenges for health-promoting interventions within municipal health and social care.

### Recruitment and Participants

To obtain rich data from a range of perspectives, a purposive sampling strategy [24] was used with the intention to include participants with experience in municipal health and social care across different organizational levels, professions, and municipalities. The inclusion criteria were that participants were employed within municipal health or social care and held one of the following roles relevant to the organization of services for older adults: occupational therapists, development managers, area managers, operations managers, or caseworkers. The initial recruitment included occupational therapists working within municipal healthcare (OT), as well as professionals in managerial or development-related roles, including development managers (DM), area managers (AM), and operations managers (OM). During the data collection process, the importance of perspectives from municipal social care became increasingly evident, and caseworkers (CW) were subsequently included, reflecting an iterative approach to sampling.

Contact was made with the heads of all municipalities (*n *= 14) in a region of northern Sweden to obtain approval for employee participation and for managers to distribute an invitation email to relevant employees. The invitation email contained information about the study, including its aim, what participation entailed, and contact details for the first author (LK). Since the head of the municipality distributed the invitation emails, the total number of potential participants reached could not be determined. Seven municipalities declined participation. In some municipalities, approval for participation was not granted by the managers, while in others no responses were received from potential participants. Follow-up contact with municipal management indicated that the invitation had been forwarded but that employees had declined participation. In both cases, nonparticipation was attributed mainly to time constraints and workload. In total, 22 participants among the seven municipalities expressed interest in participating via e-mail or telephone contact with LK and were included.

Participants were grouped with others in similar professional roles, meaning that each focus group consisted of participants operating at similar organizational levels. This was done to facilitate open discussion and reduce the potential influence of hierarchical differences within the groups. Owing to the limited number of participants in certain roles, area managers and caseworkers were included in the same focus group. This grouping was considered appropriate, as these roles operate at similar organizational levels and collaborate closely.

Five focus groups were conducted. We sought to include a variety of professions and to ensure representation from several different municipalities within the total sample, some participants within the same professional role were located far apart. Three focus groups were therefore conducted digitally because of the long distances between participants’ workplaces, and two were conducted as physical focus groups. The composition of the focus groups is presented in Table 1.

**Table 1:** Focus group compositions

Focus group	Format	Number of participants	Professional role(s)	Number of municipalities represented
1	Digital	5	Occupational therapists	2
2	Physical	5	Occupational therapists	1
3	Digital	5	Development managers	4
4	Physical	4	Area managers (*n* = 2); Case workers (*n* = *2)*	1
5	Digital	3	Operation managers	3

### Data Collection and Procedure

To prepare participants and stimulate dialogue within the focus groups, a case was used as a common point of reference for the discussions. The case, together with the question areas, was e-mailed to participants approximately 1 week prior to each focus group to support individual reflection and preparatory notetaking. As participants in the digital focus groups represented different municipalities and lacked established working relationships, and as health-promoting interventions for older adults constitute a relatively new area of practice, the case was intended to provide a shared frame of reference for discussion. The case consisted of a short description of the aim, theoretical foundation, and delivery of Health Web, a web-based health-promoting occupational therapy group intervention for older adults developed [18] and previously tested in a municipal care context [19] within our previous research. During the focus groups, the case was used as a starting point to prompt discussion, with participants encouraged to relate the question areas to the case as well as to their own experiences. The intention was not to evaluate the specific intervention, but to use it as a stimulus for broader reflection on health-promoting interventions. The question areas addressed the need for health-promoting interventions for older adults, the opportunities and challenges of working with health promotion within the municipal health and social care organization, and the potential of digital group interventions such as the one described in the case. Participants were also encouraged to move beyond the specific case in both their individual reflections and during the focus groups.

The data was collected during a 5-month period. To stimulate an open and encouraging climate, each focus group began with LK describing the aim of the study and the structure of the focus group. Furthermore, the value of participants’ thoughts and the importance of freely sharing them within the group were highlighted [24]. LK subsequently provided the participants the opportunity to ask any questions according to the case example or the related question areas that they had previously received. After written consent was obtained from the participants they began their discussions. The discussions were supported by the question areas; however, the participants were also encouraged to reflect freely on the subject. LK had the role of moderator and the fourth author (GI) was an assisting moderator. The time interval for the focus groups was between 56 min and 1 h and 50 min. Both the digital and the physical focus groups were audio recorded. LK was responsible for giving all the participants the opportunity to make their voices heard, to stimulate interaction between them, and to actively listen without interrupting the discussions. Each focus group was concluded by GI providing a short summary of the discussions on the basis of notes taken and where the participants were given the opportunity to confirm or object to whether the summarization reflected their perception of what was discussed. All focus groups were verbatim transcribed. GI took notes during all focus group discussions which included factors that could have been lost in transcribed form, such as body language, facial expressions, and tone of voice, to enrich the transcripts with a social context and the emotional tone that existed during the discussions, which can provide valuable information when analyzing the transcripts [25].

### Analysis

The analysis process was performed in accordance with the focus group analysis process described by Dahlin-Ivanoff and Holmgren [25]. The approach was inductive and guided by the study`s aim. The analysis began after each focus group was conducted, when LK and GI reflected on and noted recurring words, phrases and lines of reasoning expressed by participants. Shortly thereafter, LK listened repeatedly to the audio recordings to gain an overall sense of the material and constructed mind maps to capture recurring patterns within each focus group. These initial interpretations were then discussed between LK and GI to establish a shared understanding of the data as a whole. The verbatim transcripts were subsequently analysed in several iterative steps. GI identified meaning units related to the study aim and organized them into preliminary themes. These preliminary themes were developed through constant comparison within and across focus groups and by continuously relating the emerging interpretations back to the raw data. In line with the focus group analysis approach described by Dahlin-Ivanoff and Holmgren [25], the subthemes were developed to remain close to the participants’ shared expressions and collective reasoning. The preliminary themes reflected a descriptive level of interpretation on participants’ shared perspectives on *the need for sustained engagement* and *collaboration across organizational levels*, *unclear distribution of responsibilities and resources*, *the development of a health-promoting working culture, *and *the need for time and organizational support to enable change* and *new working methods*. These preliminary themes and their underlying interpretations were discussed among all the authors, where preliminary themes expressing similar content were merged and refined into the final subthemes. A central overarching theme was subsequently developed to capture the latent meaning across the focus groups, relating to opportunities and challenges in developing organizational readiness for health-promoting practices. The overarching theme represents a higher level of interpretation, capturing the latent meaning across the subthemes and relating to the opportunities and challenges in developing organizational readiness for health-promoting practices. In the final step of the analysis, the authors revisited the raw data to ensure that the analysis accurately reflected the participants’ discussions. Illustrative quotes were selected, condensed, and used to demonstrate the alignment between the themes and the empirical material.

## Results

**Fig. 1:**
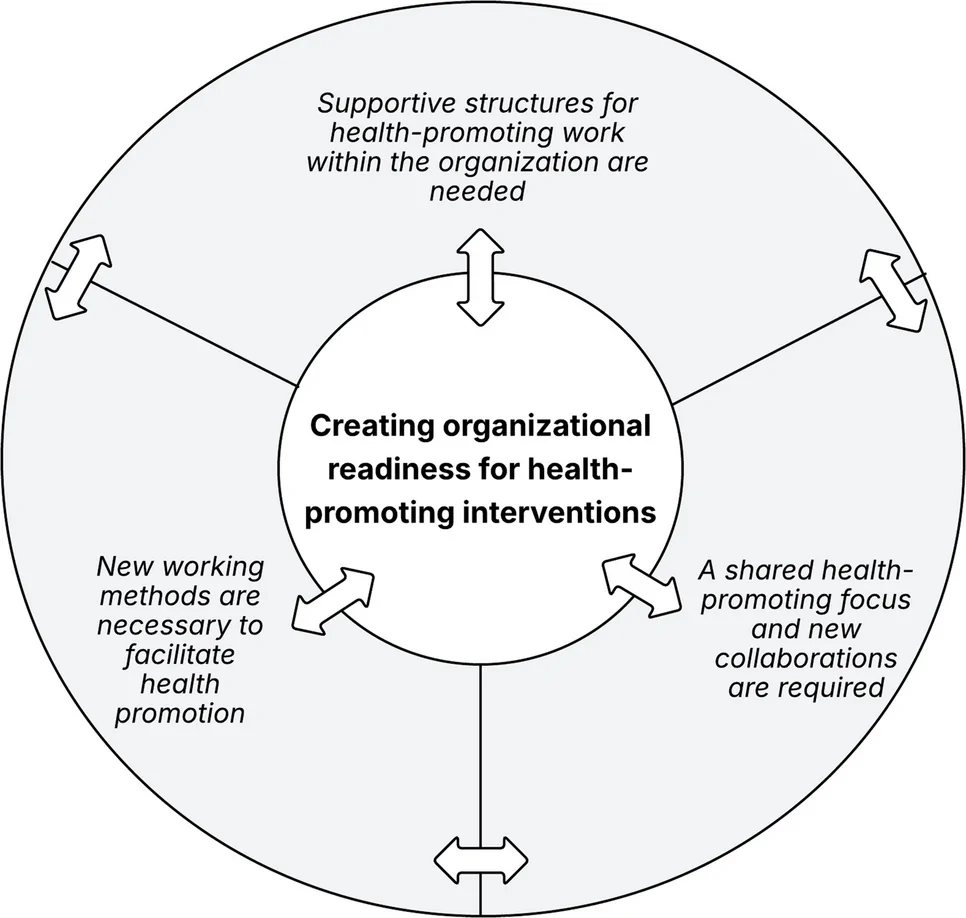
Overview of the overarching theme and subthemes

The continuous analysis resulted in one overarching theme: *Creating organizational readiness for health-promoting interventions*, which was formed by three subthemes: *Supportive structures for health-promoting work within the organization are needed*, *A shared health promotive focus and new collaborations are required*, and *New working methods are necessary to facilitate health promotion* (Fig. 1)*.*

### Creating Organizational Readiness for Health-Promoting Interventions

The overarching theme synthesized the focus group discussions, which consistently emphasized the organizational need to create readiness for health-promoting interventions for older adults. Participants in all focus groups perceived this readiness as insufficient; however, within the organization’s development for aligning with the transition to person-centered and integrated care, health-promoting interventions for older adults were regarded as essential and the need for such interventions was clearly expressed. The participants described significant challenges for individual professionals in assuming this transition. Three areas for organizational readiness towards health-promoting interventions in municipal health and social care were identified as both challenging within current practices and as offering potential opportunities for facilitation. Striving to create organizational readiness for health-promoting interventions was considered critical as part of the transition to person-centered and integrated care and gave rise to the following subthemes: *Supportive structures for health-promoting work within the organization are needed*, *A shared health promotive focus and new collaborations are required*, and *New working methods are necessary to facilitate health promotion.* The analysis revealed that these subthemes, in an interdependent and collective manner, collectively contribute to shaping organizational readiness for health-promoting interventions. The tension between recognizing the inherent opportunities for health promotion and confronting the absence of necessary conditions is reflected within the respective subthemes.

### Supportive structures for Health-Promoting Work Within the Organization are Needed

This subtheme reflects how participants described an ongoing duality between recognizing the important role of municipal healthcare within the broader healthcare system and the transition towards person-centered and integrated care and experiencing limited supportive structures for health-promoting work. Current structures, such as legal agreements, frameworks, and existing priority orders that must be adhered to, were described as not stipulating health-promoting work in regular practice, as reflected in the following reasoning among DMs in focus group 3:

*DM 1: I think that the home care agreement does not promote health-promoting group interventions because we have received funding to work with home visits […] Home visits are based on the assessment of technical aids. The focus in the Home Care Agreement is almost entirely on technical aids, and then we conduct home visits. It is not based on the person’s abilities […]*

*DM 2: Yes…*

*DM 1: I can feel that the health care agreement controls our work in that way […]*

*DM 3: yes…*

*DM 2: We do not have that responsibility in municipal health care. […] It is very regulated, and it is very directed toward a sort of treatment care and not so much preventive. […]. It’s difficult when working in it […] because one is also trained that we should work health-promoting and preventive, and that’s what we are fed with all the time. But then one becomes somewhat hindered.*

*DM 3: It’s just as you say, we are assigned to try out medical aids and what we do is just put out fires. We can never do anything to prevent the fires from occurring […] I don’t even know how one could possibly do that […] It feels difficult given how the organization and the work is structured right now anyway.*

*DM 2: Yes*

*DM 1: […] It also has a lot to do with how the agreement is structured and what assignment has been given to us in the municipality. That’s where it is, because I think, oh, to have group interventions, to have training, to send out...that looks really exciting. But where in the agreement can we...*

*DM 2: Yes*

*DM 4: Yes*

*[…]*

*DM 3: Even though we all know that it is needed and it will be needed even more.*

The lack of supportive structures for health-promoting work was described as leading to a common view of health-promoting interventions as something beyond regular practice that was optional, to take on only when time and resources were available. To enable the municipality organization to align with the ongoing transition toward person-centered and integrated care, a perceived need for revise agreements, frameworks, and priority orders was therefore expressed in all the focus groups. The new social services act, where the municipality’s responsibility for early and health-promoting measures is highlighted, was emphasized as an important step in this direction. However, a more specific interpretation of the new social services act in relation to the ongoing work and collaborations between health- and social care within the municipality organization was deemed necessary moving forward. Furthermore, the need for clarity and a concrete direction within the transition was also called for regarding the distribution of resources and how to better utilize the existing competences within the organization. This was viewed as a major work and not possible to take on by individual professionals but as something that must stem first from a political level and then be clearly expressed by management to executive professionals. The task of prioritizing and initiating the implementation of health-promoting interventions in practice was experienced as unclear and confusing in relation to today’s work situation, delimitations, and direction.

Furthermore, it was highlighted in the focus groups that these organizational changes need to be carried out from a long-term and sustainable perspective. This was, however, experienced as a challenge since long-term economic effects, as health promotion work is expected to provide, were described as difficult to measure annually. Without a clear political directive, health-promoting interventions were discussed as they might be carried out as short-term projects that could be withdrawn to meet the annual budget. Creating sustainability when health-promoting interventions were introduced was thus highlighted as a process in need of time and extra resources during the transition period, as well as a period when this work was carried out, apart from and in parallel with regular practice. It was considered too challenging and comprehensive to directly introduce health-promoting interventions into regular practice and to put the responsibility for this introduction on individual professionals.

### A Shared Health Promotive Focus and New Collaborations are Required

This subtheme reflects how it, among the focus groups, was discussed as a challenge to shift from reactive and compensatory focused interventions toward a health promotive focus. It was further discussed as a challenge that this focus shift needs to be shared on different levels in the whole organization since the professionals within municipality health and social care affect and influence each other’s work in various ways. Furthermore, shifting focus toward health promotion was described as requiring new collaborations within the organization and reaching out to collaborate with society. This was described as both a challenge and an opportunity.

It was discussed that the daily work in regular practice was permeated with a focus on reactive and focused interventions, where OTs in municipal healthcare were mainly contacted for acute, isolated compensatory interventions. This was likely due to an in-common perception of OTs as an overburdened group with few staff resources who needed to be available for recurring acute needs in connection with discharge from hospitals. For this reason, OMs, who are responsible for the OTs working conditions, for example, highlighted it as difficult to add something beyond ordinary working tasks, such as testing new health-promoting interventions. Furthermore, OTs were described as being primarily used as consultants in acute situations or when technical aid was needed. Delivering interventions within older adults’ daily activities were primarily delegated to, and carried out by, home care services. This was described as challenging, since the home care services were already an overburdened group with limited staff resources. The AMs expressed that it was therefore problematic to require their home care staff to perform delegated interventions, as they often lack formal competence and there were also many cases involving language barriers. This resulted in a work situation in which delegated interventions were frequently deprioritized in favor of basic care**.** This pervasive reactive focus within regular practice was described among the focus groups as a dissatisfactory way of working but difficult to influence at an individual level. This reasoning is illustrated in the following discussion among the OTs in focus group 1:

*OT 1: Maybe you don’t have the time to even ask about how…how is your daily life?[…] You usually think of the whole, but you don’t always have time and like that... it becomes quite a lot of these… (gesturing with a pointing hand)*

*OT 2: Yes (nodding)*

*OT 1: …isolated interventions…*

*OT 3: Yes*

*OT 1: …as one performs*

[…]

*OT 2: […] I know that I should really have time to capture it properly and work with it preventively so that we can reduce the workload later, but... it’s a bit difficult to turn the wheel when you’re already in a situation where a lot of preventive measures have been missed. Now it’s panic. Now we just need to solve this right now.*

Delivery of health-promoting interventions was discussed as a challenge as OTs were primarily described as delivering acute, isolated compensatory interventions and having consultative rather than executing roles within regular practice. Within the focus groups, this culture within the organization was discussed as resulting in distancing between OTs and resourceful older adults with ability to influence their own health. It was, however, agreed upon that a prerequisite for health-promoting interventions is to establish early contacts and map older adults’ health determinants, preferably before the onset of injury or illness. With such an approach, older adults would have greater abilities and possibilities to consciously reflect upon and fully participate in health-promoting interventions and thus may prevent or postpone poor health. Furthermore, this was discussed as an opportunity to facilitate better tailored and timely municipal healthcare interventions that may further prevent some of the acute, isolated compensatory interventions delivered within regular practice.

The discussions within the focus groups further highlighted that the collaborations between municipal health and social care are foremost brought up at a late stage in regular practice, when severe physical and/or cognitively ill functioning and complex care needs have arisen. Health determinants within older adults’ daily activities were described as not mapped by municipality social care or addressed to municipal healthcare. Thus, when shifting toward a health-promotive focus, it was suggested that municipality health and social care should collaborate more closely and foremost earlier than in regular practice. The life story form came up as a practical example for potentially being used as a tool for early mapping of health determinants, instead of as in regular practice, where the life story is filled out when moving to a nursing home. This could provide a common ground and starting point for collaboration between professionals within municipal health- and social care. Collaborative efforts between municipality health and social care were, however, considered limited and restricted by secrecy regulations, where health determinants could not automatically be referred to municipal healthcare.

Among the focus groups, resourceful older adults who experienced changes in their life situation due to aging and/or deteriorating health in themselves or others close to them were seen as most in need of health-promoting interventions. These situational changes were perceived as potential starting points that could lead to poor health and where health-promoting interventions could turn that development around. Furthermore, the process of participating in health-promoting interventions and successfully putting insights and lessons learned from these interventions into practice were discussed as demanding. However, reaching resourceful older adults capable of meeting these demands was described as a potential challenge that requires new approaches. The focus groups expressed the need for the municipal organization to position their professionals within various societal contexts and, consequently, to establish collaborations with older adults who are active and participants within these contexts. Collaborations directly with older adults themselves, within a societal context, were discussed as an opportunity for municipality health and social care to better tailor health-promoting interventions to older adults’ needs and preferences and thus become more person-centered and effective. This approach was further discussed as a way to enable older adults to become informed about available interventions and to identify what support they or others within their social circle might need to strengthen their health. This was reflected in the following discussion among the OMs in focus group 5:

*OM 1: We need to reach out to associations to a greater extent, because here in [the name of the municipality] for example, we have both neuro- and heart- and lung- and these are very active associations. […]*

*OM 2: …I think the same thing about finding these existing groups. And start spreading advertisement there about what exists […] Then it usually spreads. That I have been a part of this, and it was great, right?*

*OM 3: Yes, I would also say that it is this outreach work that is the most important for these active people to find…[…] One doesn’t really reach them otherwise than through outreach work. […]*

*OM 1: Now I started to think about what you just mentioned […] I think that a type of outreach would be…yes. […]*

*OM 2: But I think that this is really the challenge to find those people who would need it the most. Yes, but as you say, those who are part of the pensioner’s organization and the dementia association have still managed to reach a social context so that you can either reach out through them and they can talk to their acquaintances […]*

Information brochures regarding interventions given within municipality health and social care were discussed as beneficial to distribute to older adults within their first contact with the municipality, but also to distribute in various societal contexts, such as libraries and at localities for associations and interest organizations. The focus groups further discussed the need for municipality health and social care to be visible through various digital platforms where health-promoting interventions may be advertised. It was thus concluded that new collaborations with information officers within the municipalities were needed. In addition to making health-promoting interventions visible on various social media platforms, it was also deemed necessary to make the municipality’s websites more assessable and directed toward older adults.

### Facilitating Health Promotion Through New Working Methods

This subtheme reflected the focus group discussions on the group and digital format in the given case and how these formats could be adopted as working methods that may facilitate health-promoting interventions, either directly or indirectly. The incorporation of digital solutions as a work method with older adults was discussed as an opportunity to facilitate the delivery of health-promoting interventions for those who had difficulties with leaving their home. Furthermore, the focus groups emphasized the value of group dynamics within health-promoting interventions and considered it a powerful working method, as participation in activities with others was described as inherently containing health-promotive benefits. Working with groups was, however, discussed as something that had been deprioritized within regular municipal healthcare practices. Within municipal social care, examples of working with various groups were provided and suggestions emerged on how this working method could be further developed.

All the focus groups expressed a positive attitude toward keeping pace with digital development and considered incorporating digital solutions into regular practice essential. However, this was described as something currently used mainly between colleagues or for addressing organizational challenges, such as coordinating care planning meetings. The digital solutions employed in regular practice were characterized as not fostering active participation among older adults but rather providing one-way information, such as security alarms or camera surveillance for older adults with a high risk of falls. Adopting working methods that enable mutual digital communication with older adults was described as harboring the potential to increase their participation and engagement, thereby indirectly promoting health. The focus groups further reasoned that digital competence is an important skill in today’s society. Therefore, supporting older adults in developing digital competence and participation was described as an important aspect of health promotion, one that is not currently addressed within municipal healthcare and only to a limited extent within municipal social care. Furthermore, older adults were perceived as a heterogeneous group, with examples ranging from individuals interested in using digital solutions to others who were resistant to their use. Technical support for older adults was therefore discussed as something that could be aid-tested within municipal social care as a possible future task for home care services, enabling older adults’ participation in digital interventions. Adopting digital working methods was further reasoned as a tool for making health-promoting interventions more available for older adults. This was discussed as an important opportunity for those with difficulties leaving their homes, for instance, informal caregivers or older adults with limited energy and/or mobility impairments.

The need for flexibility within digital solutions was discussed among the focus groups as both an opportunity and a challenge for health-promoting interventions. For example, having digital group meetings was perceived potentially discouraging older adults from participating, yet they could also enable participation. Meeting strangers for the first time in a digital setting was considered potentially inhibiting, and combining physical introduction meetings before transitioning to digital meetings was suggested as a facilitating modification. However, for some older adults, meeting via a screen could feel safer. It was reasoned that for those who were or had been isolated in their homes for various reasons, breaking isolation by physically attending larger group meetings could be challenging. Sitting at home in front of a screen when meeting others in a larger group could lower the threshold for these individuals and serve as a first step toward eventually breaking their physical isolation. The group format itself, both digitally and physically delivered, was considered valuable and health-promoting. Regardless of the group’s purpose, content, or composition, engaging in activities with others was described as involving indirect health-promoting factors. Having a structured schedule with a set time and a place to meet others was described as stimulating cognitive, social, emotional, and physical functioning. It was further described as giving greater meaning to the performance of daily activities, such as taking care of one’s physical appearance. The group format was also discussed as creating a sense of coherence and community, which could promote mental health. This reasoning was reflected in the following discussion among the AM’s and CW’s in focus group 4:

*AM 1: We love belonging and doing things together. [...] The wife of one of our patients said, “My husband, it had been so long since I had seen him strive to look good, and he pulled himself together and became a completely different person, gained a completely different self-image because he looked forward to meeting other people.” So that really stuck with me. […] The fact that you get to meet other people, doing this in a group is so important. It gives you extra energy and the results are much better and faster...*

*AM 2: Yes, I can compare it a little bit with these carer groups. [...] there are many who are alone at home, but after a while you see how much they get out of just sharing... so I think it’s important to continue with these group meetings...*

*CW 1: In our professional role, we also see many people who are alone at home and who find it difficult to get around... things have happened in their lives, a spouse has passed away, relatives have their own health problems and can’t get around to visit. So, I think there is a need for health promotion initiatives for them, and I also think, as you mentioned, that a group is a great way to do that...*

In the discussions, examples were given of various group activities for older adults previously offered within municipal health and social services, such as balance training groups and cooking groups. These group activities were experienced as providing a context for general health-promoting conversation, information, advice, and support that may facilitate healthy lifestyle habits. However, the group activities that were highlighted as good examples were gradually phased out within municipal healthcare to reduce organizational costs. Some activities, such as support groups for home care were still provided by municipal social care but had, in some municipalities, been developed into meeting places for open activities. These meeting places were discussed as valuable health-promoting contexts for groups of older adults, which could be further expanded and more explicitly focused on health promotion. For instance, the focus group suggested that these places could be utilized for new working methods, such as delivering health-promoting lectures and information to groups of resourceful older adults.

## Discussion

This study explored how professionals across multiple roles and organizational levels in municipal health and social care discuss opportunities and challenges related to health-promoting interventions for older adults, providing insights grounded in perspectives close to everyday practices. The results suggest that these opportunities and challenges are not static but reflect an ongoing tension that shapes organizational readiness for health-promoting interventions, which both enable and constrain the transition towards person-centered and integrated care [3]. Taken together, the organizational readiness within municipal health and social care was perceived as something that has inherent potential to be actively created, but at present, it is insufficient for health-promoting interventions to permeate practice. These results are aligned with and help to contextualize the follow-up report from the National Board of Health and Welfare [26]. Although the transition towards person-centered and integrated care has been ongoing for several years, the report indicates that it has had a limited impact on practice and that key goals remain unmet [26].

Organizational readiness has been defined as stemming from the degree of change efficacy and change commitment among the individuals within the organization [27]. The results of this study indicate that within municipal health and social care, there is an unclear division of responsibilities for health-promoting work, ambiguous perceived agency, and a perceived lack of capacity to carry out health-promoting interventions due to high workloads. In terms of organizational readiness, this could be described as low change efficacy and fragmented change commitment among the individuals within the organization [27]. However, the results extend this conceptualization by showing that organizational readiness is not merely a shared psychological state, but is continuously shaped through structural conditions, shared views, and collaborative practices embedded in everyday work processes. Thus, the results show that creating readiness within the municipal organization is a complex process that requires changes in regular practices through continuous development at various organizational levels. These results align with the consolidated framework for implementation research (CFIR), which conceptualizes implementation as influenced by multiple contextual domains, including organizational structures, external conditions, individual characteristics, and implementation processes [28]. The subthemes identified in this study, highlighting supportive structures, shared health-promotive focus, collaborations, and new working methods, reflect several of the domains within the CFIR [28]. However, the qualitative approach of this study provides further depth through the participants’ reasonings showing that these are not static but are dynamically interrelated in everyday practice. Rather than acting as isolated barriers or facilitators, they interact and give rise to ongoing tensions that shape the conditions for implementing health-promoting interventions. This aligns with previous critiques of organizational readiness in healthcare, highlighting that readiness is often conceptualized inconsistently and, in many cases, is reduced to either individual attitudes or structural conditions, without sufficiently capturing the dynamic interplay between these dimensions [29]. Thus, this study contributes to a nuanced understanding of organizational readiness within municipal health and social care by showing that organizational readiness emerges as a continuously negotiated process, shaped through structural conditions, collaborative practices, and everyday work processes.

First, the need for supportive structures reflects how current legal, organizational, and economic conditions constrain the implementation of health-promoting interventions. Consistent with previous research [30–32], the implementation of health-promoting interventions was perceived as challenging, particularly in contexts characterized by insufficient organizational support, unclear roles, and a lack of coordinated structures. Furthermore, the results show that health-promoting interventions were discussed as short-term projects rather than integrated into regular practice, which may hinder sustainability [33]. These results thus reflect how structural conditions contribute to the tension between policy ambitions and the reality of everyday work practices. Furthermore, consistent with previous research [31, 34], the results highlight the importance of leadership in facilitating change, particularly in complex healthcare contexts. Consequently, this study may provide a foundation for dialogue aimed at bridging the gap between policymakers and leaders with operational responsibilities, as highlighted as needed in the follow-up report [26]. It also underscores the importance of involving occupational therapists in such dialogues, particularly in discussions concerning how their competencies and resources can be most effectively utilized within the organization.

Second, the need for a shared health-promotive focus and new collaborations illustrates how shared understandings and collaborative practices are central to organizational readiness, shaping how responsibility for health-promoting interventions is perceived and enacted in everyday practice. This aligns with previous research highlighting the importance of individual readiness and learning cultures for successful organizational change [35]. Furthermore, collaborative efforts between researchers and those who deliver and receive interventions play a vital role in the development of new health-promoting interventions [33]. Future research should therefore embrace collaborative co-creation processes [36], which can drive the development and implementation of new interventions [33]. While the study captures shared experiences and reasoning focused on separate organizational levels, the results highlight the need for increased interaction between these levels. This study thus provides a foundation for future collaborative co-creation processes [36], aimed at developing and facilitating organizational readiness for change towards more health-promoting practices. This may also imply a need for dialogue regarding where within the organization occupational therapy competencies can be most effectively positioned to support health-promoting interventions.

The results indicate that the group format was perceived as inherently containing health-promoting effects for older adults. Participation in group-based interventions was described as fostering mutual encouragement and shared learning, where individuals influenced and motivated each other in ways that strengthened their sense of agency. Engaging with others who shared similar experiences and aspirations was perceived to enhance empowerment, as participants were able to support one another beyond what could be achieved through individual professional guidance alone. Furthermore, preparing to participate in group activities was described as meaningful in itself, providing a sense of purpose in everyday life, such as taking care of one’s appearance and preparing to meet others. These reasonings align with qualitative research on social and relational processes among older adults in group-based settings, which highlights how shared experiences, mutual support, and social learning processes contribute to increased self-efficacy and the development of agency [37]. In this study, as in previous research [19, 38], digital solutions were regarded by professionals as having the potential to reach individuals who, for various reasons, find it difficult to participate in traditional, in-person interventions. The results further suggest that digitally delivered group interventions may offer opportunities to combine the benefits of group dynamics with more accessible and resource-efficient modes of delivery. However, digital solutions were described as primarily used for communication between professionals rather than as tools for interaction with older adults or for delivering interventions. This indicates a need to further develop how digital solutions can be integrated into health-promoting practices directed at older adults, particularly in ways that retain the relational and motivational benefits of group-based approaches. Variation in older adults’ motivation to use digital solutions was identified in this study’s results as a key challenge for digital group interventions. Additionally, in line with previous research [39], the results raised concerns about older adults’ potential need for technical support to enable participation in such interventions. Access to and responsibility for providing such support emerged as major challenges for introducing new digital health-promoting group interventions within municipal health and social care. These challenges further reflect the tensions identified in this study, where the potential of digital solutions to enhance health promotion is constrained by the practical conditions of everyday practices and varying levels of digital competence among older adults. This also highlights that, while occupational therapists have established experience in using group-based approaches as therapeutic mechanisms, there is a need for dialogue on how digital solutions can be meaningfully integrated within the profession to support health-promoting practices among older adults.

Taken together, this study highlights the need for collaborative co-creation approaches across different levels within organizations. The identified subthemes may serve as a basis for structured dialogue between policymakers, managers, and professionals, aimed at jointly exploring how challenges can be addressed and opportunities strengthened. Combining qualitative findings with a structured implementation framework [28] within a comprehensive approach can support a shift towards coordinated, multifaceted efforts that enable more systemic health-promoting practices within municipal health and social care.

## Methodological Considerations

The use of focus groups enabled the generation of rich data, capturing a breadth of perspectives across professional roles and organizational levels within municipal health and social care. However, the results should be interpreted in light of several methodological limitations. To facilitate transferability [24], detailed information about sampling, participant characteristics, data collection, and analysis is provided in the methods section. As recruitment relied on municipal management to disseminate the invitation e-mail, the authors had limited control over its distribution. This may have introduced a degree of gatekeeper influence on participation. However, follow-up communication with the municipal management also indicated that high workload and limited resources lead to not forwarding the invitation e-mail and also for potential participants declining. The literature [25] emphasizes the importance of homogeneity within each group to explore shared experiences. To achieve homogeneity, participants with similar professional responsibilities were grouped together and placed in the same focus group. Additionally, in the focus groups held in place, all the participants worked within the same municipality. Conducting profession-specific focus groups allowed for the inclusion of diverse perspectives across organizational levels in the overall analysis while maintaining homogeneity within each focus group. This approach facilitated open discussions, aiming to enable participants to draw on shared experiences and develop collective reasoning within their professional context. Although grouping intended to reduce hierarchical constraints, power dynamics may still have influenced how participants expressed their views. The use of both digital and in-person focus groups may have influenced group interaction and dynamics. Digital formats can facilitate participation across geographical distances but may limit non-verbal communication and spontaneity in discussions. At the same time, using both formats enabled the inclusion of participants from multiple municipalities, which strengthened the diversity of perspectives in the study. Trustworthiness [24] was strengthened by ensuring that all the focus groups were conducted under similar conditions and that the data were collected collaboratively by two of the authors. The first author (LK) has prior professional experience in municipal healthcare. Although such a preunderstanding poses a risk of bias, it may also enrich and deepen the analysis by providing contextual insight. This duality illustrates a common challenge in qualitative research: balancing the benefits of insider knowledge with the need for reflexivity and transparency [24]. The potential influence of preunderstanding has been reduced since participants were recruited from multiple professional roles, organizational levels, and municipalities with no prior connection to the first author. Within the research group are senior researchers in occupational therapy with complementary expertise. Together, the research group has extensive experience with ageing research, with a focus on social participation, health promotion, and digital interventions. Additionally, the research group has extensive experience in qualitative research, including focus group methodology. Data collection and analysis were conducted collaboratively within the research team, supporting reflexivity and rigor throughout the process. Throughout the analysis, interpretations were continuously discussed within the research team to critically reflect on how preunderstanding may have influenced the analysis.

The use of a predefined case may have introduced framing bias by directing participants’ reflections towards a specific type of intervention. The case was, however, intentionally used as a stimulus to facilitate discussion, particularly as participants did not know each other and represented different municipalities within a relatively new area of practice. While participants were encouraged to reflect beyond the case, it cannot be excluded that it influenced the discussions. The topics addressed are, however, relevant beyond the specific case and reflect broader conditions within municipal health and social care. Furthermore, the authors’ prior involvement in the development of the intervention presented within the case entails a preunderstanding that may have influenced both data collection and interpretation, requiring ongoing reflexivity. Additionally, by using the participants’ own words in illustrative quotes, this contributed to credibility by reducing the risk that the material is influenced by the author’s own preconceptions or ideas [24, 25].

## Conclusions and Implications for Occupational Therapy

The identified needs for supportive structures, a shared focus on health promotion, and new working methods reflect interconnected conditions that were described as both constraining in current practice and offering potential opportunities for facilitation. Taken together, these results indicate that organizational readiness within municipal health and social care is currently insufficient for health-promoting interventions to permeate practice yet has inherent potential to be actively created. Creating such readiness therefore requires coordinated, long-term efforts across the organization, rather than relying on individual professionals or isolated initiatives.

For occupational therapy, these results underscore the importance of engaging in ongoing dialogue, both within the profession and across organizational levels, on how more proactive and health-promoting approaches can be translated into everyday practice. While occupational therapists hold competencies highly relevant for health promotion, their ability to act on these competencies is shaped by prevailing organizational conditions and reactive practices. This highlights the need not only to engage in dialogue, but also to actively contribute to how these competencies are utilized in practice, including critically examining the balance between reactive and more health-promoting approaches. Such considerations may involve rethinking how occupational therapy is positioned within municipal health and social care, including closer integration with social care contexts.

## Data Availability

The datasets generated and analyzed during the current study are not publicly available to protect the participants’ confidentially (in accordance with the European Union’s General Data Protection Regulation) but are available from the corresponding author upon reasonable request.

## References

[1] Stucki G, Bickenbach J, Gutenbrunner C, et al. Rehabilitation: the health strategy of the 21st century. J Rehabil Med. 2018;50(4):309–16. 10.2340/16501977-2200. 28140419

[2] World Health Organization. World report on ageing and health. Geneva (Switzerland): World Health Organization; 2015.

[3] European Commission. Green Paper on Ageing: fostering solidarity and responsibility between generations. COM(2021) 50 final. [Internet]; 2021. Available from: https://commission.europa.eu/system/files/2021-06/green_paper_ageing_2021_en.pdf. Accessed 7 Sep 2026.

[4] Regeringskansliet. Hälso- och sjukvårdslag (SFS 2017:30) [Health and Medical Services Act]. [Internet]; 2017. Swedish. Available from: https://www.riksdagen.se/sv/dokument-och-lagar/dokument/svensk-forfattningssamling/halso-och-sjukvardslag-201730_sfs-2017-30/. Accessed 19 May 2026.

[5] Socialstyrelsen. Hälso- och sjukvård i hemmet: kunskapsstöd för personcentrerad vård och rehabilitering [Health and medical care at home: knowledge support for person-centred care and rehabilitation]. [Internet]; 2023. Swedish. Available from: https://www.socialstyrelsen.se/contentassets/010b67e7a23a47dbaacb99a8ff77188e/2023-3-8458.pdf. Accessed 19 Nov 2025.

[6] Sveriges kommuner och regioner. Primärvård i Sverige: uppdrag, organisation, kontinuitet och prioriteringar [Primary care in Sweden: mission, organization, continuity and priorities]. [Internet]; 2024. Swedish. Available from: https://extra.skr.se/download/18.f2b28b1199ffd2ba2f8be59/1762593064942/SKR_A4_Primarvardsrapporten_2024_webbpdf.pdf. Accessed 19 May 2026.

[7] Beard JR, Hanewald K, Si Y, et al. Cohort trends in intrinsic capacity in England and China. Nat Aging. 2025;5(1):87–98. 10.1038/s43587-024-00741. 39702725 PMC11754101

[8] Thorvaldsson V, Karlsson P, Skoog J, et al. Better cognition in new birth cohorts of 70 year olds, but greater decline thereafter. J Gerontol B Psychol Sci Soc Sci. 2017;72(1):16–24. 10.1093/geronb/gbw125. 27974472

[9] Thorvaldsson V, Skoog I, Johansson B. Delayed onset of cognitive terminal decline in later born cohorts: evidence from a longitudinal study of two cohorts born 29-years apart. Psychol Aging. 2024;40(1):109–16. 10.1037/pag0000846. 39235909

[10] Statistics Sweden. After age 60: a description of older people in Sweden. Demographic reports. [Internet]; 2022. Swedish. Available from: https://www.scb.se/contentassets/c4ac9fb5ad10451aab0885b7160de9b0/be0701_2022a01_br_be51br2202.pdf. Accessed 5 Mar 2026.

[11] Nutbeam D, Muscat DM. Health promotion glossary 2021. Health Promot Int. 2021;36(6):1578–98. 10.1093/heapro/daaa157. 33822939

[12] Bajraktari S, Sandlund M, Zingmark M. Health-promoting and preventive interventions for community-dwelling older people published from inception to 2019: a scoping review to guide decision making in a Swedish municipality context. Arch Public Health. 2020;78(1):97. 10.1186/s13690-020-00498-9. 33072316 PMC7556574

[13] Kantartzis S. The Dr Elizabeth Casson memorial lecture 2019: shifting our focus. Fostering the potential of occupation and occupational therapy in a complex world. Br J Occup Ther. 2019;82(9):553–66. 10.1177/0308022619864893.

[14] World Federation of Occupational Therapists. About occupational therapy. [Internet]; 2025. English. Available from: https://wfot.org/about/about-occupational-therapy. Accessed 5 Mar 2026.

[15] Regeringskansliet. Sveriges digitaliseringsstrategi 2025–2030 [Sweden's digitalization strategy 2025–2030]. [Internet]; 2025. Swedish. Available from: https://www.regeringen.se/contentassets/fe3e296228fb474f803a986ae3842b4c/sveriges-digitaliseringsstrategi-20252030.pdf. Accessed 28 May 2026.

[16] Zingmark M, Fisher AG, Rocklöv J, et al. Occupation-focused interventions for well older people: an exploratory randomized controlled trial. Scand J Occup Ther. 2014;21(6):447–57. 10.3109/11038128.2014.927919. 25022428

[17] Behm L, Eklund K, Wilhelmson K, et al. Health promotion can postpone frailty: results from the RCT elderly persons in the risk zone. Public Health Nurs. 2016;33(4):303–15. 10.1111/phn.12240. 26568469

[18] Nyman A, Larsson E, Isaksson G. Health web 1.0, a web-based occupational therapy group intervention for older adults: a study protocol for a feasibility study. Scand J Occup Ther. 2025;32(1):2590725. 10.1080/11038128.2025.2590725. 41255086

[19] Körlof L, Larsson E, Isaksson G, et al. Experiences of a digital group intervention ‘the health web’: a case study of older adults. Scand J Occup Ther. 2025;32(1):2477115. 10.1080/11038128.2025.2477115. 40104972

[20] Socialstyrelsen. SOSFS 2011:9 Ledningssystem för systematiskt kvalitetsarbete [Management systems for systematic quality work]. [Internet]; 2011. Swedish. Available from: https://www.socialstyrelsen.se/contentassets/8c944a90bf4741d28be747285a69b76e/2011-6-38.pdf. Accessed 19 May 2026.

[21] Socialstyrelsen. Kommuners förutsättningar att arbeta hälsofrämjande och förebyggande i samverkan [Municipal conditions for health promotion and disease prevention in collaboration]. [Internet]; 2025. Swedish. Available from: https://www.socialstyrelsen.se/globalassets/sharepoint-dokument/dokument-webb/ovrigt/kommuners-forutsattningar-halsoframjande-och-forebyggande.pdf. Accessed 19 May 2026.

[22] Moore GF, Audrey S, Barker M, et al. Process evaluation of complex interventions: Medical Research Council guidance. BMJ. 2015;350:h1258. 10.1136/bmj.h1258. 25791983 PMC4366184

[23] O’Cathain A, Croot L, Duncan E, et al. Guidance on how to develop complex interventions to improve health and healthcare. BMJ Open. 2019;9(8):e029954. 31420394 10.1136/bmjopen-2019-029954 PMC6701588

[24] Holloway I, Galvin K. Qualitative research in nursing and healthcare. Chichester: John Wiley & Sons; 2023.

[25] Dahlin-Ivanoff S, Holmgren K. Fokusgrupper: greppbar metod [Focus groups: tangible method]. 1st ed. Lund: Studentlitteratur; 2017. Swedish.

[26] Socialstyrelsen. Uppföljning av omställningen till en god och nära vård – delredovisning 2025 [Follow-up on the transition to good and close care – interim report 2025]. [Internet]; 2025. Swedish. Available from: https://www.socialstyrelsen.se/contentassets/e5373180cfe042f7877af6dc9fecd386/2025-9-9707.pdf. Accessed 26 Nov 2026.

[27] Weiner BJ. A theory of organizational readiness for change. Implement Sci. 2009;4:67. 10.1186/1748-5908-4-67. 19840381 PMC2770024

[28] Damschroder LJ, Aron DC, Keith RE, et al. Fostering implementation of health services research findings into practice: a consolidated framework for advancing implementation science. Implement Sci. 2009;4:50. 19664226 10.1186/1748-5908-4-50 PMC2736161

[29] Caci L, Nyantakyi E, Blum K, et al. Organizational readiness for change: A systematic review of the healthcare literature. Implement Res Pract. 2025;6:26334895251334536. 40385227 10.1177/26334895251334536 PMC12084713

[30] Dohrn IM, von Berens Å, Olsson CB, et al. Between principles and pragmatism–primary healthcare and social services professionals’ experiences and perceptions of self-care for older adults with home care: a qualitative study. Scand J Prim Health Care. 2025;43(1):36–46. 10.1080/02813432.2024.2389116. 39126195 PMC11834817

[31] Moore L, Britten N, Lydahl D, et al. Barriers and facilitators to the implementation of person‐centred care in different healthcare contexts. Scand J Caring Sci. 2017;31(4):662–73. 10.1111/scs.12376. 27859459 PMC5724704

[32] Strömqvist Bååthe K, Aytar O, von Thiele SU, et al. Art of developing a vision for integrated healthcare and social services: an interview study with stakeholders in Sweden. J Integr Care. 2024;32(5):135–48. 10.1108/JICA-08-2024-0044.

[33] Skivington K, Matthews L, Simpson SA, et al. A new framework for developing and evaluating complex interventions: update of medical research council guidance. BMJ. 2021;374:n2061. 10.1136/bmj.n2061. 34593508 PMC8482308

[34] Moore J, Elliott IC, Hesselgreaves H. Collaborative Leadership in Integrated Care Systems; Creating Leadership for the Common Good. J Change Manag. 2023;23(4):358–73. 10.1080/14697017.2023.2261126.

[35] Choi M, Ruona WE. Individual readiness for organizational change and its implications for human resource and organization development. Hum Resour Dev Rev. 2011;10(1):46–73. 10.1177/15344843103849.

[36] Agnello DM, Anand-Kumar V, An Q, et al. Co-creation methods for public health research—characteristics, benefits, and challenges: a Health CASCADE scoping review. BMC Med Res Methodol. 2025;25(1):60. 10.1186/s12874-025-02514-4. 40050729 PMC11884017

[37] Duplantier SC, Hayes MG, Sanchez-Zaragoza N, et al. Community as medicine: a qualitative study of how group health coaching and social connection improve mental well-being in older adults. Healthcare (Basel). 2026;14(4):510. 10.3390/healthcare14040510. 41754023 PMC12940478

[38] Barchéus IM, Ranner M, Månsson Lexell E, et al. Occupational therapists’ experiences of using a new internet-based intervention - a focus group study. Scand J Occup Ther. 2024;31(1):2247029. 10.1080/11038128.2023.2247029. 37708913

[39] Ge H, Li J, Hu H, et al. Digital exclusion in older adults: a scoping review. Int J Nurs Stud. 2025;168:105082. 10.1016/j.ijnurstu.2025.105082. 40279791

